# Supplementary material for: Awareness Regarding Antimicrobial Resistance and Antibiotic Prescribing Behavior among Physicians: Results from a Nationwide Cross-Sectional Survey in India
Source: Antibiotics (Basel). 2023 Sep 29;12(10):1496. doi: 10.3390/antibiotics12101496 (PMC10604884; doi:10.3390/antibiotics12101496)
Supplement: Supplementary file 1 [file antibiotics-12-01496-s001.zip › antibiotics-2604754-supplementary.pdf]

STUDY QUESTIONNAIRE

Awareness regarding Antimicrobial resistance and antibiotic prescribing behaviour among physicians

Name: Age/gender:

Highest educational qualification: MBBS/ MD/ DM/ MCh

Speciality/ Super-speciality:

Type of work setting: Government/ Private / Charitable organization

Type of setting – PHC/CHC/District Hospital/Tertiary care hospital/Private Hospital/Private clinic/Others

Name of the Government Institute/ Hospital presently working with:

Official designation: Senior resident/ Assistant Professor/ Associate Professor/ Additional Professor/ Professor/ Consultant

Years of practice after postgraduation:

Please answer the following questions:

KNOWLEDGE

Q1. Indiscriminate use of antibiotics in humans, plants and animals leads to antimicrobial resistance. YES/ NO/ DON'T KNOW

Q2. Approximately 30 percent of all hospitalised patients recieve antibiotics at any given time. YES/ NO/ DON'T KNOW

Q3. Lack of rapid diagnostic tests is one of the reasons for irrational antibiotic use. YES / NO/ DON'T KNOW

Q4. Limited access to essential antibiotics contributes to irrational antibotic use and emergence of antibiotic resistance. YES/ NO/ MAYBE

Q5. Broad spectrum antibiotics, when used inappropriately, lead to emergence of antibiotic resistance. YES/ NO/ DON'T KNOW

Q6. Are you familiar with the WHO AWaRe classification of antibiotics? YES / NO

Q7. Antibiograms for different hospitals in a region are usually similar. YES / NO/ DON'T KNOW

Q8. Hospital antibiograms serve as important tools in guiding empiric antibiotic therapy and tracking resistance patterns. YES / NO/ DON'T KNOW

Q9. As per your knowledge, which is the most prescribed antibiotic in COVID-19 pandemic? (Please tick appropriately)

- Azithromycin
- Doxycycline
- Co Amoxyclav
- Cefixime
- Any other, please specify

Q10. Which of the following agents are effective against infections by anaerobes?

- Metronidazole
- Meropenem
- Co-amoxyclav
- All of the above

Q11. When clinically desirable, the route of administration of antibiotics may be switched from intravenous to oral (IV to oral switch) due to the following reason/s:

- Decreases the duration of hospitalisation
- More convenient for patient
- More time consuming in adult patients
- Lesser complications

ATTITUDE

Q12. Do you think that your antibiotic prescribing behavior has an impact on the development of antibiotic resistance in your region? YES / NO/ DON'T KNOW

Q13. According to you, who among the following can play a key role in addressing the issue of antibiotic resistance? (can tick more than one)

- Doctors/ prescribers
- Patients/ consumers
- Nursing/ paramedical staff
- Community as a whole
- Government or Regulatory authority
- Pharmacists/ Chemists
- All of the avove

Q14. In your opinion, which of the following strategies can help in addressing the issue of antibiotic resistance?

- Shorter duration of antibiotic therapy
- Reduced non-prescription sale of antibiotics

- Infection control measures e.g. hand hygiene, cohorting etc.
- Antibiotic use restricted to cases with confirmed bacterial infections

Q15. How much do you agree to prescribing antibiotics on patients' demands?

- Strongly agree
- Agree
- Neither agree nor disagree
- Disagree
- Strongly disagree

Q16. Surveillance of antibiotic use and resistance should be done regularly at hospital, local, regional, national and global levels to combat antimicrobial resistance.

- Strongly agree
- Agree
- Neither agree nor disagree
- Disagree
- Strongly disagree

Q17. There is a rampant use of antibiotics in the hospital I am working.

- Strongly agree
- Agree
- Neither agree nor disagree
- Disagree
- Strongly disagree

PRACTICES

Q18. Which sources of information do you mostly use while making decisions on antibiotic prescription?

- Treatment guidelines
- Conferences/ Trainings/ CMEs
- Journals/ Text books
- Internet/Social media
- Expert opinions
- Other (please specify)

Q19. Which factor/s mostly determine your choice of antibiotics?

- Culture susceptibility reports
- Availability of antibiotics
- Cost of antibiotics

- Local resistance patterns
- Recommendations from seniors or colleagues
- Recommendations from pharmaceutical companies
- Own experience

Q20. Do you ever employ delayed/ back-up antibiotic prescribing in your clinical practice? YES/ NO/ DON'T KNOW

Q21. How frequently do you counsel the patients regarding appropriate use of antibiotics to prevent emergence of resistance?

- Never
- Rarely
- Sometimes
- Always

Q22. How often do you change the empiric antibiotic prescribed on the basis of culture sensitivity report?

- Never
- Rarely
- Sometimes
- Always

Q23. How often do you discontinue the empiric antibiotic in case of negative culture report?

- Never
- Rarely
- Sometimes
- Always

Q24. How much do you prefer prescribing two or more class/es of antibiotics in combination over single agents?

- Never
- Rarely
- Sometimes
- Always

Q25. How often do you prescribe antibiotic/s prophylactically without evidence of infection?

- Never
- Rarely
- Sometimes
- Always

Q26. During last 12 months, did you attend any trainings/ conferences to update your knowledge on antibiotic use? YES/ NO
